# Interleukin 37 limits monosodium urate crystal-induced innate immune responses in human and murine models of gout

**DOI:** 10.1186/s13075-016-1167-y

**Published:** 2016-11-18

**Authors:** Lei Liu, Yu Xue, Yingfeng Zhu, Dandan Xuan, Xue Yang, Minrui Liang, Juan Wang, Xiaoxia Zhu, Jiong Zhang, Hejian Zou

**Affiliations:** 1 Department of Rheumatology, Huashan Hospital, Fudan University, 12# Wulumuqi Road, Jingan District, Shanghai, China; 2 Institute of Rheumatology, Immunology and Allergy, Fudan University, 12# Wulumuqi Road, Jingan District, Shanghai, China; 3 Department of Pathology, North Huashan Hospital, Fudan University, 108# Luqiang Road, Baoshan District, Shanghai, China

**Keywords:** IL-37, MSU, Gout, Smad3, IL-1R8, NLRP3, Mertk, S​OCS3

## Abstract

**Background:**

Interleukin (IL)-37 has emerged as a fundamental inhibitor of innate immunity. Acute gout is a self-limiting inflammatory response to monosodium urate (MSU) crystals. In the current study, we assessed the preventive and therapeutic effect of recombinant human IL-37 (rhIL-37) in human and murine gout models.

**Methods:**

We investigated the expression of IL-37 in patients with active and inactive gouty arthritis and assessed the effect of rhIL-37 in human and murine gout models: a human monocyte cell line (THP-1) and human synovial cells (containing macrophage-like and fibroblast-like synoviocytes) exposed to MSU crystals, a peritoneal murine model of gout and a murine gouty arthritis model. After inhibition of Mer receptor tyrosine kinase (Mertk), levels of IL-1β, IL-8 and chemokine (C-C motif) ligand 2 (CCL-2) were detected by ELISA and expression of mammalian homologs of the drosophila Mad gene 3 (Smad), suppressor of cytokine signaling 3 (SOCS3), NACHT-LRR-PYD-containing protein 3 (NLRP3), and IL-8R of THP-1 were assessed by qPCR and western blot to explore the molecular mechanisms.

**Results:**

Our studies strongly indicated that rhIL-37 played a potent immunosuppressive role in the pathogenesis of experimental gout models both in vitro and in vivo, by downregulating proinflammatory cytokines and chemokines, markedly reducing neutrophil and monocyte recruitment, and mitigating pathological joint inflammation. In our studies, rhIL-37 suppressed MSU-induced innate immune responses by enhancing expression of Smad3 and IL-1R8 to trigger multiple intracellular switches to block inflammation, including inhibition of NLRP3 and activation of SOCS3. Mertk signaling participated in rhIL-37 inhibitory pathways in gout models. By inhibition of Mertk, the anti-inflammatory effect of rhIL-37 was partly abrogated, and IL-1R8, Smad3 and S​OCS3 expression were suppressed, whereas NLRP3 expression was reactivated.

**Conclusions:**

Our studies reveal that IL-37 limits runaway inflammation initiated by MSU crystal-induced immune responses, partly in a Mertk-dependent fashion. Thus, rhIL-37 has both preventive and therapeutic effects in gouty arthritis.

## Background

Interleukin (IL)-37 is a member of the IL-1 family, discovered by computational cloning, and previously termed IL-1 family member 7 [1]. Quite distinct from most IL-1 family members, which are characterized by proinflammatory functions, IL-37 has emerged as a fundamental inhibitor of the innate immune response by shifting the cytokine equilibrium away from excessive inflammation [2]. The function of IL-37 is also caspase-1-dependent. Caspase-1 processing is required not only for maturation of the intracellular IL-37 precursor and translocation of the cytokine to the nucleus, but also for the secretion of mature IL-37. IL-37 is reported to be a dual-function cytokine with intracellular and extracellular properties. IL-37 interacts intracellularly with mammalian homologs of the drosophila Mad gene 3 (Smad3), whereas the IL-1R8–IL-18Rα complex acts as the cell surface receptor for IL-37, and IL-37 uses IL-1R8 to trigger multiple intracellular switches to block inflammation [3, 4].

Gout is a typical innate immune response triggered by monosodium urate crystal (MSU); IL-1β is critical to the acute gouty inflammation [5], while transforming growth factor β1 (TGF-β1) plays an important role in limiting inflammation [6]. As IL-37 functions as a natural suppressor of innate immunity, whether and how IL-37 downregulates inflammation induced by the danger signal MSU is not known. Although IL-37 exhibits the opposite function to IL-1β, there are many similarities between them: first, they are both first synthesized as precursor molecules and are processed to their mature forms by NACHT-LRR-PYD-containing protein 3 (NLRP3)-activated caspase-1 cleavage [3]; second, toll-like receptor (TLR) ligands can induce their intracellular precursor expression and cause inflammasome priming. IL-37 shares the same intracellular inhibitory conduction signal Smad3 with the immunoregulatory cytokine TGF-β1. Interestingly, IL-1β and low concentrations of TGF-β1 have been shown effective in inducing endogenous IL-37, which is relevant to the interaction of IL-37 with Smad3 [2]. Based on these findings, we speculated that upon stimulation by MSU, macrophages produce not only the proinflammatory cytokine IL-1β, but also the anti-inflammatory cytokine IL-37 through NLRP3 activation, to initiate a negative feedback mechanism to curb excessive inflammation.

In the current study, we investigated the expression of IL-37 in patients with active and inactive gouty arthritis. We also assessed the effect of recombinant human IL-37 (rhIL-37) in different human and murine gouty models: a human monocyte cell line (THP-1), human synovial cells (containing macrophage-like and fibroblast-like synoviocytes), a peritoneal murine model of gout and a murine gouty arthritis model. Our results support the idea that the presence of rhIL-37 attenuates the inflammatory process in gout, and it has both preventive and therapeutic effects. To our knowledge, this is the first study to show the anti-inflammatory properties of rhIL-37 in human and murine gouty models.

## Methods

### Patients and healthy controls

A total of 31 patients with gout were included in the current study. The classification of gout fulfilled the 1977 American Rheumatism Association (now the American College of Rheumatology (ACR)) preliminary criteria for the classification of the acute arthritis of primary gout and the 2015 ACR/European League Against Rheumatism (EULAR) gout classification criteria [7, 8]. Gouty patients were divided into acute (*n* = 16) and non-acute (*n* = 15) groups. Healthy controls (*n* = 18) with no hyperuricemia, no metabolic syndrome or other chronic diseases were recruited. Peripheral blood samples were obtained from patients with gout and healthy controls. Serum samples were stored at −80 °C until cytokine levels were determined. Peripheral blood mononuclear cells (PBMCs) from patients and healthy controls were isolated using Ficoll-Paque PLUS (GE Healthcare, Piscataway, NJ, USA), according to the manufacturer’s instructions. The collected cells were used for cell cultures or stored at −80 °C until RNA extraction. Synovial fluids were collected from five patients with acute gout and stored at −80 °C prior to assay. Synovial tissue from patients with active gouty arthritis and subcutaneous tophus from patients with chronic tophaceous gout were subjected to immunohistochemical analysis.

### Preparation of MSU crystals

MSU crystals were prepared as described by Denko and Whitehouse [9]. Briefly, 4 g uric acid was dissolved in 800 ml of deionized water, heated to 60 °C, adjusted to pH 8.9 with 0.5 N NaOH, and crystallized overnight at room temperature. MSU crystals were recovered by centrifugation, washed with distilled water and dried at 40 °C for 24 h. Crystal shape and birefringence were assessed by compensated polarized light microscopy. MSU crystals were milled and then sterilized by heating at 180 °C for 2 h before each experiment. We measured <0.015 EU/ml endotoxin in MSU crystal preparations using a Limulus amebocyte lysate assay (Sigma-Aldrich, St Louis, MO, USA).

### Induction of gout models in vitro and rhIL-37/Mertk inhibitor treatment

#### THP-1-derived macrophages

THP1 monocytes were suspended in complete medium, consisting of RPMI 1640, 2 mM L-glutamine, 100 units/ml of penicillin, 100 μg/ml of streptomycin, supplemented with 10% fetal bovine serum (FBS) (Gibco BRL, Grand Island, NY, USA) and were seeded in 24-well culture plates (2 × 10^5^ cells/ml/well). Cells then were treated with 50 ng/ml phorbol myristate acetate (PMA) for 24 h to generate THP-1-derived macrophages [2]. THP-1-derived macrophages were treated with or without 10 ng/ml recombinant human IL-37 (rhIL-37; R&D Systems, Minneapolis, MN, USA) for 3 h, followed by incubation for 1 h with or without a small-molecule inhibitor of Mertk (Mertk inhibitor UNC2250, 20 nM; Selleckchem, Houston, TX, USA) and then incubated with either 1 μg/ml lipopolysaccharide (LPS) (Sigma), 5 mM ATP (Sigma), or MSU (50 μg/ml, 100 μg/ml) separately for a further 18 h. Culture supernatants were harvested and frozen at −80 °C for later cytokine analysis by ELISA.

#### Synoviocytes

Synovial tissue specimens were obtained from patients with osteoarthritis, who were undergoing surgical joint replacements. Synovial cells (containing macrophage-like and fibroblast-like synoviocytes) were isolated from tissue explants and cultured as previously described [10]. Briefly, tissues were minced and incubated with a solution containing 0.15 mg/ml DNase, 0.15 mg/ml hyaluronidase (type I-S), and 1 mg/ml collagenase (type IA) (Sigma-Aldrich) in Dulbecco’s modified Eagle’s medium (DMEM; Invitrogen, Carlsbad, CA, USA) for 1 h at 37 °C. Cells were washed and resuspended in DMEM supplemented with 10% FBS, 30 mg/ml glutamine, 250 μg/ml amphotericin B (Sigma-Aldrich), and 20 μg/ml gentamicin (Invitrogen). After overnight culture, non-adherent cells were removed, and adherent cells were cultured and seeded in 6-well culture plates (1 × 10^6^ cells/ml/well).

All experiments were performed with cells obtained after passage 2. Cell-specific markers were used to identify synoviocyte populations. Macrophage-like synoviocytes (MLS) were CD68^+^, as demonstrated by specific fluorescence antibody staining (Biolegend, San Diego, CA, USA) and flow cytometry analysis. Synovial cells were treated with or without rhIL-37 (R&D Systems) 10 ng/ml for 3 h and incubated with LPS 1 μg/ml, ATP 5 mM, or MSU (50 μg/ml, 100 μg/ml) separately for a further 6 h or 18 h. The supernatants were harvested and frozen at −80 °C for later cytokine analysis by ELISA.

### In vivo experiments and IL-37/Mertk inhibitor treatment

#### MSU crystal-induced murine peritonitis

Male C57BL/6 mice, 6–8 weeks old, were injected intraperitoneally (IP) with MSU crystals suspended in phosphate-buffered saline (PBS) (3 mg/0.5 ml). rhIL-37 (1000 ng/mouse) was given IP once either 72 h, 48 h and 24 h prior to MSU crystal administration or 1 h after it. All controls received vehicle alone. At 3 h after MSU crystal administration, mice were killed and monitored for cell recruitment in the peritoneal lavage fluid, and chemokine expression and production. Cells collected by peritoneal lavage were retained for subsequent flow cytometry analysis. Peritoneal lavage fluid was stored at −80 °C for later chemokine analysis by LEGENDplex™ Muti-Analyte Flow Assays (Biolegend).

#### Murine gouty arthritis

Male C57BL/6 mice, 6–8 weeks old, were anesthetized in by inhalation of 3% isoflurane (Forane; Abbot, Chicago, IL, USA) and then intra-articular injected with 50 μl (1 mg) MSU suspension into the right foot pad [11]. PBS was injected into the left foot pad at the same time as a control. rhIL-37 was given IP once either 72 h and 24 h prior to MSU crystal administration or 1 h and 4 h after it and at different concentrations (1000 and 100 ng/mouse). For the Mertk inhibitor group, UNC2250 (60 μg UNC2250/100 μl dimethyl sulfoxide (DMSO)) was injected IP into mice prior to MSU crystal administration and rhIL-37 intervention on two successive days. All controls received the diluent alone. At 8 h after MSU crystal administration, mice were killed after monitoring for joint swelling. Joint tissues were removed and stored for further RNA and protein extraction, or fixed in formalin for histopathologic analysis.

#### Foot thickness evaluation

Joint swelling was examined by digimatic caliper (Mitutoyo, Kawasaki, Japan), the minimum accuracy of which was 0.01 mm [12]. Joint inflammation was expressed as the ratio of the foot pad thickness of the inflamed over the normal control joint; values exceeding 1.10 were categorized as inflammation.

#### Histological studies and immunohistochemistry

Mouse joint tissue sections were prepared and stained with H&E. Synovial tissue from patients with active gouty arthritis and subcutaneous tophus from patients with chronic tophaceous gout were subjected to immunohistochemical analysis. Rabbit anti-IL-37 antibody (ab153889; Abcam, Cambridge, MA, USA) and goat anti-rabbit IgG H&L (ab97051; Abcam) secondary antibody were used. Each sample was incubated with an isotypic antibody dilution under the same experimental conditions as the negative control. Images were acquired using a light microscope (Olympus, Tokyo, Japan) with × 10 and × 20 objectives, and analyzed with image processing software (Olympus).

### ELISA

The levels of cytokines or chemokines in serum or supernatants were measured by ELISA (IL-1β, TGF-β1, IL-8 and chemokine (C-C motif) ligand 2 (CCL2) ELISAs were from R&D Systems; IL-37 ELISA was from Cloud Clone Corp®, Houston, TX, USA). The detection limits of the ELISAs were <4.8 pg/ml for IL-1β, <15.4 pg/ml for TGF-β1, <7.5 pg/ml for IL-8, <10 pg/ml for CCL2 and <3.1 pg/ml for IL-37, respectively.

### Multi-analyte flow assays

The levels of proinflammatory chemokines in mouse peritoneal lavage fluid were measured by LEGENDplex™ Multi-Analyte Flow Assays (Biolegend) Mouse Proinflammatory Chemokine Panel (13-plex, Cat. No. 740007). Methods were performed as recommended by the manufacturer.

### Fluorescence staining and flow cytometry analysis

For fluorescent staining of cell surface markers, mouse peritoneal cells were stained with fluorescent antibodies to the surface markers F4/80, Gr-1, and clone 7/4 (F4/80 and Gr-1 were from Biolegend; clone 7/4 was from AbD Serotec, Kidlington, UK) then washed and resuspended in fluorescence-activated cell sorting (FACS) buffer. Peritoneal cell populations were sorted based on their expression of F4/80, Gr-1, and clone 7/4. Monocytes were Gr-1^inter^ 7/4^+^ F4/80^low^, neutrophils were Gr-1^high^ 7/4^+^ F4/80^-^ and macrophages with high expression of F4/80 (gate R4) were thought to be resident macrophages [13]. FACS was performed using the Accuri C6 (BD Biosciences, San Jose, CA, USA).

### Real-time PCR and qPCR-array

Total RNA was extracted with Trizol (Invitrogen, Carlsbad, CA, USA) according to the manufacturer’s instructions. cDNAs were prepared using the iScript™ cDNA Synthesis kit (Bio-Rad, Hercules, CA, USA). PCR primers (BioTNT, Shanghai, China) used for RT-PCR were as follows: human pro-IL-37: forward 5′-AGTGAGGTCAGCGATTAGGA-3′ and reverse 5′-TTTTAGTGAGCAGGTTTGGT-3′; human β-actin: forward 5′-CCACGAAACTACCTTCAACTCC-3′, and reverse 5′-GTGATCTCCTTCTGCATCCTGT-3′. RNA samples were normalized to control housekeeping genes (human β-actin) and relative mRNA levels of target genes were calculated by the 2^-ΔΔ^ cycle threshold (Ct) method.

For PCR array experiments, an RT2 Profiler Custom PCR array was used to simultaneously examine the mRNA levels of 89 genes, including five housekeeping genes in 96-well plates according to the protocol of the manufacturer (SABiosciences, QIAGEN, Hilden, Germany). Real-time PCR was performed using the RT2 SYBR green qPCR Master Mix (SABiosciences) on an ABI 7500 Fast 96-well real-time PCR machine (Applied Biosystems, Foster City, CA, USA). Results were analyzed using the PCR Array Data Analysis Web Portal (SABiosciences). Glyceraldehyde-3-phosphate dehydrogenase (GAPDH) and β-actin were chosen as internal loading controls for standardization between samples and relative mRNA levels of target genes were calculated by the 2^-ΔΔ^ Ct method.

### Western blot analysis

Equal amounts of whole joint tissue were lysed in lysis buffer and tissue proteins extracted. Protein levels in different groups were expressed as a ratio to that of the corresponding GAPDH. We used rabbit polyclonal anti-mouse NLRP3 antibody, anti-mouse Smad3, anti-mouse IL-1R8, anti-mouse S​OCS3 (all antibodies were from Santa Cruz Biotechnology, Dallas, TX, USA) and rabbit polyclonal anti-mouse GAPDH antibody (Cell Signaling Technology, Danvers, MA, USA) as primary antibody and anti-rabbit IgG HRP-linked antibody (Cell Signaling Technology) as secondary antibody.

### Statistical analysis

Experiments were performed at least three times. Data were analyzed with GraphPad Prism 5.01 (GraphPad Software, La Jolla, CA, USA) and are presented as the mean (± S.E.M) or median (range). Repeated measures analysis of variance (ANOVA) followed by the Student Newman-Keuls (S-N-K) test were used for post-hoc analysis of differences between groups. *P* values <0.05 were considered to indicate statistically significant differences.

## Results

### Expression of IL-37 in patients with gouty arthritis

**Fig. 1:**
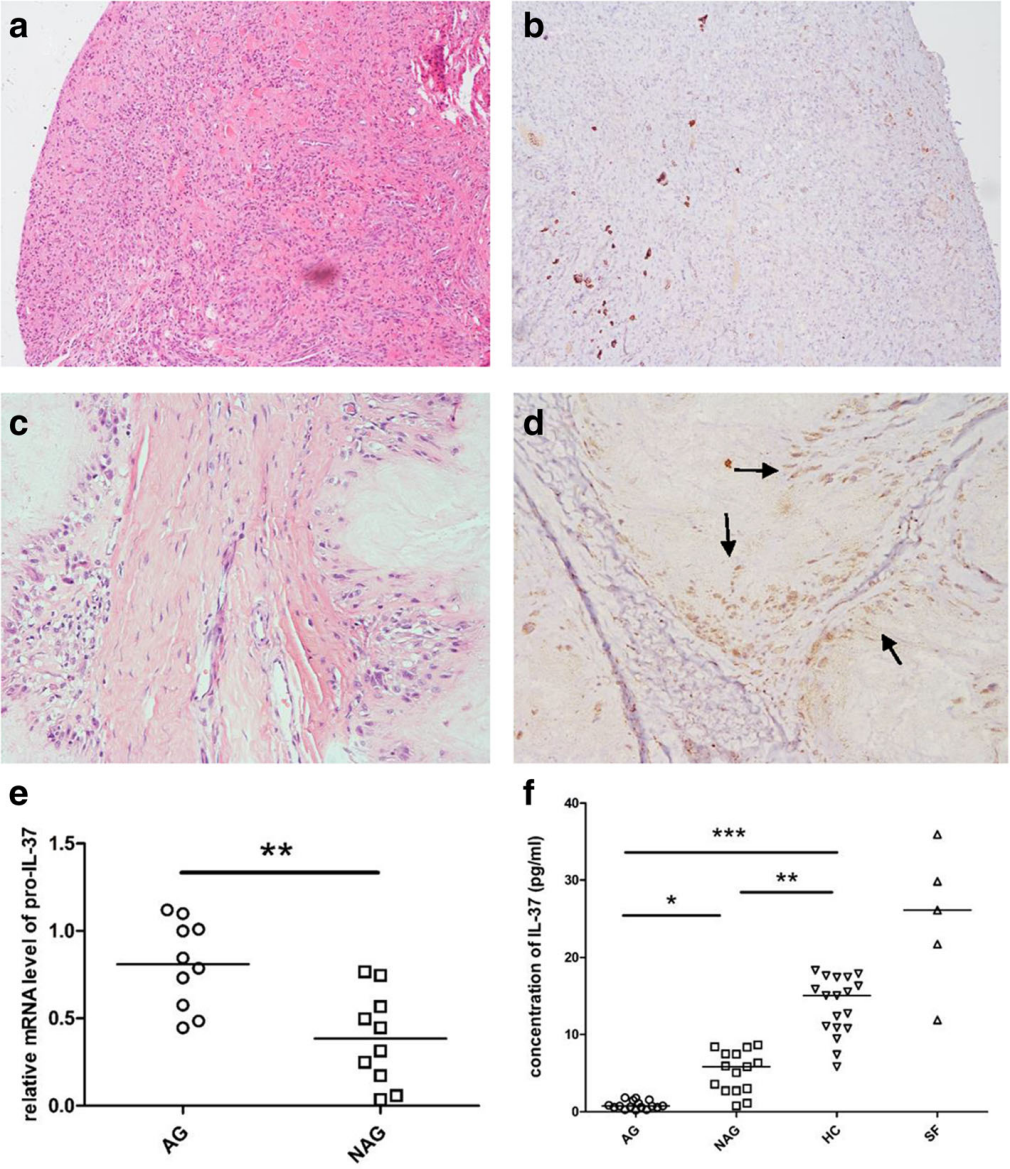
Expression of IL-37 in patients with gouty arthritis. **a** H&E staining of synovial tissue from a person with active gouty arthritis. **b** Immunohistochemical staining of IL-37 in the same synovial tissue. **c** H&E staining of subcutaneous tophus from patients with chronic tophaceous gout. **d** Immunohistochemical staining of IL-37 (*arrow*) in the same subcutaneous tophus. **e** Concentration of IL-37 in serum from patients with acute gout (*AG*, n = 16) or non-acute gout (*NAG*, n = 15), and from healthy controls (*HC*, n = 18), and in synovial fliud (*SF*) from patients with acute gout (n = 5); *P < 0.05,**P < 0.01,***P < 0.001. **f** Expression of pro-IL-37 mRNA in the peripheral blood mononuclear cells (PBMCs) from patients with gout (*AG*, n = 10, *NAG*, n = 10); **P < 0.01

Diseased synovial lining from patients with active gouty arthritis contained small amounts of IL-37 (Fig. 1a, b), whereas tissue around chronic tophus from patients with chronic tophaceous gout contained large amounts of IL-37 (Fig. 1c, d). IL-37 was highly expressed in sera from patients with non-acute gouty arthritis and in synovial fluid from patients with acute gouty arthritis (Fig. 1e), and the mRNA level of pro-IL-37 in periperhal blood mononuclear cells (PBMC) from patients with acute gouty athritis was much higher than that from patients with non-acute gouty arthritis (Fig. 1f).

### IL-37 is inducible in the PBMCs stimulated by MSU

**Fig. 2:**
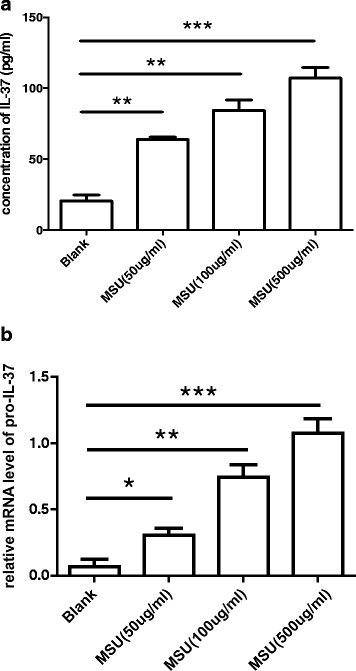
IL-37 is inducible in the peripheral blood mononuclear cells (PBMCs) stimulated by monosodium urate (*MSU*). **a** Concentration of IL-37 protein in PBMC after MSU stimulation for 18 h; **P < 0.01,***P < 0.001. **b** The expression of pro-IL-37 mRNA in the PBMCs after MSU stimulation for 18 h; *P < 0.05,**P < 0.01,***P < 0.001

Previous studies have shown that IL-37 is inducible in PBMCs by various TLR ligands [2]. Thus, we investigated whether endogenous IL-37 could be directly induced by MSU crystals as a danger signal in PBMCs. To address the question, freshly isolated PBMCs were treated with different concentrations (50, 100, and 500 μg/ml) of MSU crystals for 18 h and we discovered that both protein and mRNA expression of IL-37 was increased dose dependently upon stimulation with MSU (Fig. 2a, b).

### IL-37 abrogates proinflammatory cytokines in THP-1 macrophages and human synoviocytes

As macrophages, especially resident macrophages, were demonstrated to play a pivotal role in the initiation of acute gouty inflammation and the subsequent inflammatory cascade [14], we assessed whether the inflammation induced by MSU in macrophages could be dampened by IL-37 in vitro. We used THP-1 monocyte-derived macrophages and synovial cells containing macrophage-like synoviocytes, which were identified as resident macrophages in joints [15].

**Fig. 3:**
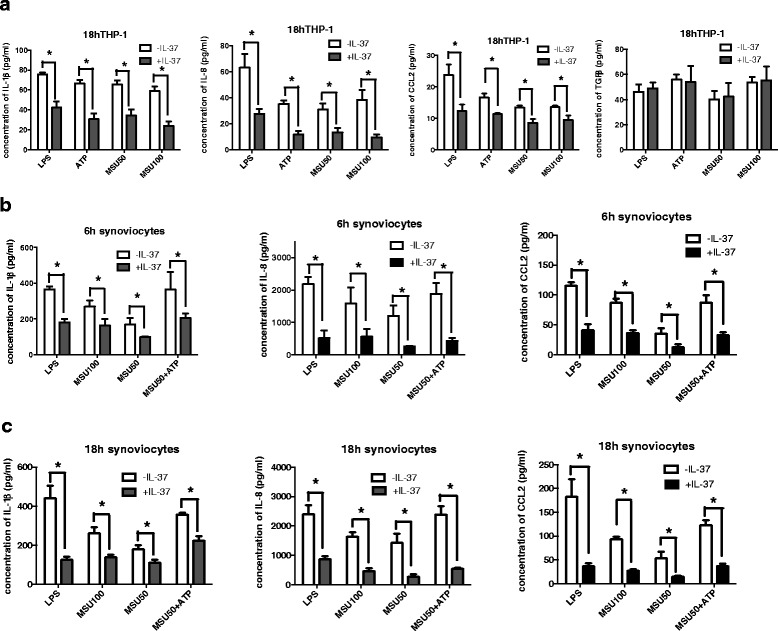
Recombinant human IL-37 (*rhIL-37*) treatment in THP-1 macrophages and human synoviocytes. **a**–**c** Concentration of secreted IL-1β, IL-8, chemokine (C-C motif) ligand 2 (CCL2) and transforming growth factor β (TGF-β) in synoviocytes or THP-1 left untreated or treated with rhIL-37 for 3 h, and then incubation with monosodium urate (*MSU*), ATP or lipopolysaccharide (*LPS*) for a further 6 h or 18 h. Mean ± s.e.m. *P < 0.05 for rhIL-37 treatment compared to non-rh-IL-37 treatment

Treatment with rhIL-37 consistently resulted in reducing the production of mature IL-1β, IL-8 (CXCL8) and CCL2 in both THP-1 macrophages (Fig. 3a) and synoviocytes (Fig. 3b, c) stimulated with MSU, ATP or LPS. In contrast, the expression of the anti-inflammatory cytokine TGF-β (Fig. 3a) in the same supernatants of THP-1 macrophages was unaffected by treatment with rhIL-37. The basal production of mature IL-1β, IL-8 (CXCL8) and CCL2 in THP-1 and synoviocytes under rhIL-37 treatment was below the minimum detection limitation (data not shown).

### IL-37 limits the innate immune response in mice with MSU crystal-induced peritonitis

We then assessed the impact of IL-37 on an MSU-crystal-induced mouse peritonitis model. rhIL-37 (1000 ng/mouse) was given IP once either 72 h, 48 h and 24 h prior to administration of MSU crystals or 1 h after it. We observed potent preventive and therapeutic immunosuppressive effects over 3 h in the peritoneal model, and the effects on inflammatory cell recruitment and related chemokines were evaluated.

**Fig. 4:**
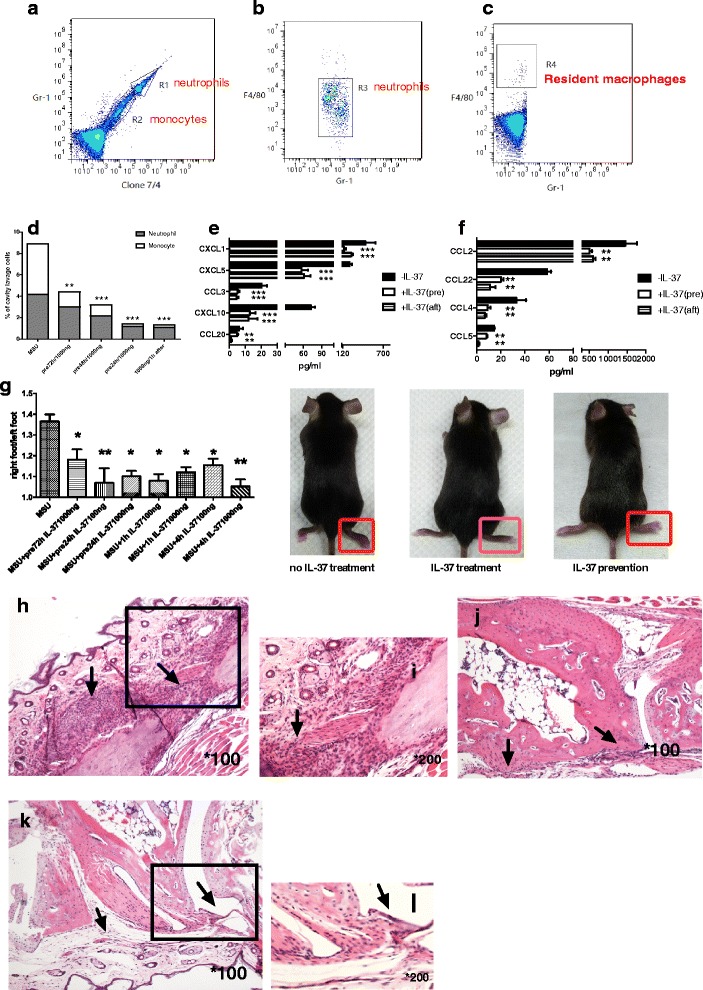
Recombinant human 1 L-37 (*rhIL-37*) reduces inflammatory cell recruitment in mice with monosodium (*MSU*) crystals induced peritonitis. **a**–**c** Neutrophil, monocyte and macrophage determination by flow cytometry: neutrophils are defined as Gr-1^high^7/4^+^ (gate *R1*); monocytes are defined as Gr-1^inter^7/4^+^ (gate *R2*) expressing low levels of F4/80 (gate *R3*); macrophages with high expression of F4/80 (gate *R4*) are thought to be resident macrophages. **d** Total and separate proportions of neutrophils and monocytes in all peritoneal exudate cells; **P < 0.01,***P < 0.001. **e** Concentration of neutrophil chemoattractant in peritoneal lavage fluid; ***P < 0.001. **f** Concentration of monocyte chemoattractant in peritoneal lavage fluid; **P < 0.01. **g** Different dosage of rhIL-37 was given preventively or therapeutically in mice with gouty arthritis, and foot thickness was evaluated; **P < 0.01, ***P < 0.001. **h**, **i** Histopathological analysis of joint from non-rhIL-37 treatment group by H&E staining (×100 original magnification (**h**) and × 200 original magnification (**i**)); *arrow* abundant inflammation in soft tissue and joint space. **j** Histopathological analysis of joint from rhIL-37 preventive group by H&E staining (×100 original magnification); *arrow* less inflammation in soft tissue and joint space. **k**, **l** Histopathological analysis of joint from rhIL-37 therapeutic group by H&E staining (×100 original magnification (**k**) and × 200 original magnification (**l**); *arrow* almost no inflammation in soft tissue and joint space

Peritoneal exudate cells were sorted by flow cytometry based on their expression of F4/80, Gr-1, and clone 7/4. Monocytes were determined as Gr-1^inter^ 7/4^+^ F4/80^low^, while neutrophils were determined as Gr-1^high^ 7/4^+^ F4/80^-^ [13] (Fig. 4a–c). rhIL-37 given before or after MSU crystal administration markedly diminished (55–75%) neutrophil and monocyte influx into the peritoneal cavity (Fig. 4d).

We tested an array of 13 mouse proinflammatory chemokines. In peritoneal cavity fluid, the production of the major neutrophil chemoattractants CXCL1 (KC), CXCL10 (IP-10), and CCL3 (MIP-1α) was almost completely suppressed (82–88%, Fig. 4e). Similarly, the monocyte chemoattractants CCL2 (MCP-1), CCL5 (RANTES), CCL4 (MIP-1β), and CCL22 (MDC), and the additional neutrophil chemoattractants CXCL5 (LIX) and CCL20 (MIP-3α) were considerably reduced (51–69%, Fig. 4e, f). The mean changes in other chemokines CCL11 (Eotaxin), CCL17 (TARC) and CXCL9 (MIG) were also lower, whereas interestingly, CXCL13 (BLC) was increased (data not shown).

Thus, these data indicate that rhIL-37 has potent preventive and therapeutic effects in vivo by abrogating the expression of proinflammatory chemokines, markedly reducing neutrophil and monocyte recruitment in MSU-induced peritoneal inflammation.

### IL-37 attenuates MSU-induced arthritis in mice

We also assessed the impact of IL-37 on an MSU crystal-induced mouse arthritis model. rhIL-37 was given IP once either 72 h and 24 h prior to MSU crystal administration or 1 h and 4 h after it and at different concentrations (1000 and 100 ng/mouse). Mice were killed 8 h after MSU crystal injection and the right paws were removed to assess the effects on foot thickness and joint histopathological change. Amelioration of foot swelling was observed whenever treated with rhIL-37 at either dosage, before or after MSU crystal administration (Fig. 4g). However, the optimal effect was shown when rhIL-37 was given at a dosage of 1000 ng, 4 h after MSU crystal injection (Fig. 4g). The result is consistent with foot histopathological findings. HE staining in the non-rhIL-37 treatment group revealed marked inflammatory cell infiltration in the subcutaneous soft tissue and joint space (Fig. 4h, i). In contrast, in the rhIL-37 prevention groups there was significantly less inflammatory cells infiltration (Fig. 4j). These effects were also shown in the IL-37 treatment group by H&E staining, in addition to the visible tissue damage and vascular hemorrhage caused by the injection, and inflammatory cells were difficult to find (Fig. 4k, l).

These results suggest that IL-37 exerts both sufficiently preventive and therapeutic effects on MSU-induced arthritis. To our surprise, IL-37 was a potent anti-inflammatory agent, even given at 4 h after MSU crystal administration (Fig. 4g), at which time point the inflammatory cascade was shown to have started in the mouse gouty arthritis model in our previous study [16].

### IL-37 enhances Smad3, IL-1R8, S​OCS3 and Mertk expression, whereas it suppresses NLRP3 expression

**Fig. 5:**
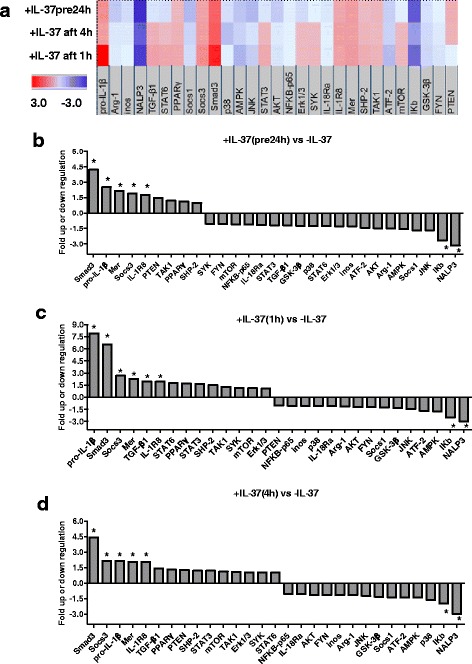
Underlying mechanism involved in recombinant human IL-37 (*rhIL-37*) inhibitory properties. **a** Heat map of the mRNA qPCR-array analysis. *Color intensity* is proportional to the fold-regulation, which represents fold-change results in a biologically meaningful way. Fold-regulation values greater than 1.5 indicate positive or up-regulation (*red*), whereas values less than -1.5 indicate negative or downregulation (*blue*). With fold-change values greater than one the fold-regulation is equal to the fold-change.) Fold-change values less than one indicate negative or downregulation, and the fold-regulation is the negative inverse of the fold-change. Fold-change is expressed as a ratio by 2^-ΔΔct^ in the rhIL-37 intervention group/2^-ΔΔct^ in the non-rhIL-37 intervention group. **b**–**d** Fold-regulation in different rhIL-37 intervention groups vs the non-rhIL-37 intervention group, respectively; *P < 0.05

**Fig. 6:**
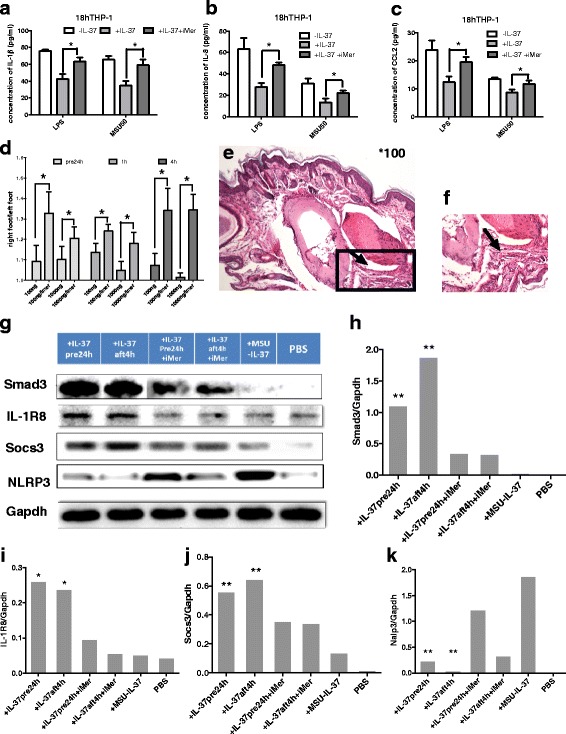
Contribution of the Mertk inhibitor to the IL-37-mediated anti-inflammatory effect in monosodium urate (*MSU*)-induced models in vitro and in vivo. **a**–**c** Concentration of secreted IL-1β, IL-8 and CCL2 in THP-1 macrophages treated with or without recombinant human IL-37 (rhIL-37) for 3 h, followed by incubation for 1 h with or without Mertk inhibitor and then incubated with lipopolysaccharide (LPS) or MSU separately for a further 18 h; *P < 0.05. **d** Different dosage of rhIL-37 was given preventively or therapeutically with or without Mertk inhibitor intervention in mice with gouty arthritis, and foot thickness was evaluated; *P < 0.05. **e**, **f** Histopathological analysis by H&E staining in a joint from the group treated with rhIL-37 treatment and Mertk inhibitor intervention (×100 original magnification (**e**) and × 200 original magnification (**f**); *arrow* inflammation in soft tissue and joint space. **g**–**k** The protein level of Smad3, IL-1R8, S​OCS3 and NLRP3 was verified by western blotting in the IL-37 treatment groups with or without Mertk inhibitor intervention. Protein levels in different groups were expressed as a ratio to that of corresponding glyceraldehyde-3-phosphate dehydrogenase (*GAPDH*); *P < 0.05 **P < 0.01

To investigate the underlying mechanism involved in the inhibitory properties of rhIL-37, qPCR-array and western blotting were carried out in the murine acute gouty arthritis model. First, total RNA from experimental joints was extracted and the qPCR-array carried out to evaluate the mRNA expression of IL-37-related signal transduction molecules. The mRNA levels of pro-IL-1β, Smad3, IL-1R8, S​OCS3 and Mertk were significantly upregulated in the IL-37 intervention groups, whereas the mRNA expression of NLRP3 was significantly downregulated (fold change >1.5 and *P* < 0.05, Fig. 5a–d). The protein level of Smad3, IL-1R8, S​OCS3 and NLRP3 was then verified by western blotting, and the results indicated that IL-1R8, Smad3 and S​OCS3 were highly expressed, whereas NLRP3 was suppressed in the IL-37 intervention groups (*P* < 0.05, Fig. 6g–k).

### Inhibition of Mertk partly reduced IL-37-mediated anti-inflammatory effects in MSU-induced models in vitro and in vivo

In the murine acute gouty arthritis model, the mRNA level of Mertk was significantly upregulated in the IL-37 intervention groups. Thus, we conducted further studies to confirm the contribution of Mertk to IL-37-mediated inflammation inhibition by blocking its activity with the small-molecule inhibitor UNC2250 in MSU-stimulated THP-1 macrophages pretreated with rhIL-37 and in the murine gouty model treated with rhIL-37. Inhibition of Mertk reduced the IL-37-mediated inhibition of inflammation, resulting in upregulation of IL-1β, IL-8 and CCL2 in the supernatant of THP-1 macrophages exposed to MSU (Fig. 6a–c). The basal production of mature IL-1β, IL-8 (CXCL8) and CCL2 in THP-1 under MERTK inhibitor treatment was below the minimum detection limit (data not shown).

UNC2250 was injected intraperitoneally into mice prior to MSU crystal administration and IL-37 intervention for two successive days. In the arthritis model, exacerbated foot thickness and aggravated pathological digitoplantar inflammation were also observed in the Mertk inhibition groups (Fig. 6d–f). Further western blotting analysis identified that IL-1R8, Smad3 and S​OCS3 were suppressed, whereas NLRP3 expression was increased in the Mertk inhibition groups (Fig. 6g–k). These findings suggest that IL-37-mediated inhibition of inflammation is partly dependent on Mertk activation.

## Discussion

Acute gout is an inflammatory response to MSU crystals in the joints and periarticular tissue. An interesting feature of acute gout is the self-limiting nature of the inflammatory flare. The inhibition of this inflammatory response is linked to a number of regulatory events ranging from crystal coating and apoptotic cell clearance, through to proinflammatory cytokine regulation and TGF-β1 production. However, current research into the pathways involved in the resolution of inflammation remains limited. IL-37 has emerged as a fundamental inhibitor of the innate immune response by shifting the cytokine equilibrium away from excessive inflammation. IL-37 has been shown to attenuate inflammation in models of septic shock, chemical colitis, cardiac ischemia and contact dermatitis [17–21]. Based on the above findings, we are greatly interested in whether and how IL-37 downregulates inflammation induced by MSU in gouty arthritis.

First, IL-37 was demonstrated to be expressed in synovial tissue and chronic tophus in patients with gouty arthritis. Diseased synovial lining contained small amounts of IL-37, whereas tissue around chronic tophus contained large amounts of IL-37. Thus, we hypothesized that IL-37 might be involved in the limitation of MSU-induced inflammation because in chronic tophus, abundant crystals do not always trigger inflammation. We also found that serum IL-37 was obviously higher in healthy controls (HC) than in patients with non-acute gout (NAG) and patients with acute gout (AG) (HC > NAG > AG). The results suggest that the expression of IL-37 is closely related to the internal inflammatory state. An elevated level of inflammation is associated with lower serum IL-37. High IL-37 in HC and NAG may protect individuals from inflammation. Furthermore, we found that the mRNA level of pro-IL-37 in PBMCs from patients with AG was much higher than that from those with NAG, which suggests that inflammation may promote pro-IL-37 mRNA expression in PBMCs to curb the excess inflammation. Accordingly, we investigated if MSU could directly induce the expression of IL-37 in PBMCs. We stimulated PBMCs with different concentrations of MSU and discovered that both protein and mRNA expression of IL-37 was increased dose dependently. Based on these findings, we thus verified our speculation that upon stimulation by MSU, macrophages produced not only the proinflammatory cytokine IL-1β, but also the anti-inflammatory cytokine IL-37. In future studies, a time-course of IL-37 vs IL-1β production would be informative to determine whether MSU-induced IL-37 is a downstream effect of MSU-induced IL-1β production (or TLR activation).

Second, different gouty arthritis models were used to investigate the inhibitory effect of IL-37 in vitro and in vivo. As IL-37 was identified in the diseased synovial tissue from patients with gouty arthritis, synovial cells containing both MLS and FLS were exposed to MSU in studies in vitro. Our previous study revealed that exposure of FLS to MSU crystals only transiently induced an increase in IL-1β expression, which was apparently not sufficient to initiate the inflammatory cascade as seen in resident macrophages [22]. Therefore, besides observing the inhibitory effect of rhIL-37 in vitro, we also hypothesized that MLS may play the role of resident macrophages to initiate inflammation in gouty arthritis. Our results showed that rhIL-37 was prominent in suppressing the production of IL-1β, IL-8, and CCL2 in THP-1 macrophages and synovial cells stimulated with MSU, and this anti-inflammatory effect was TGF-β independent.

We also confirmed our previous hypothesis that MLS could play a key role in the initiation of the inflammatory cascade upon MSU stimulation by releasing major proinflammatory cytokines and chemokines in joints to recruit neutrophil and monocyte infiltration. Accumulating evidence indicates that chemokines, which are powerful leukocyte chemoattractants and activators, contribute to acute gout inflammation [23]. Recent studies have shown that MSU crystals induce FLS to release CCL2, thus, diminishing CCL2 production may limit the inflammatory process and monocyte recruitment in joints [24]. The recruitment and activation of neutrophils is central to acute gouty inflammation. The CXC family of chemokines plays an important role in neutrophil chemotaxis. Indeed, increased levels of CXCL8 (IL-8), CXCL1 (KC) and CXCL10 (IP-10) have been detected in synovial fluid in patients with gouty arthritis [25, 26]. In our in vivo studies, rhIL-37 consistently exerted an effective inhibitory role in decreasing neutrophil and monocyte infiltration by dramatically reducing the expression of major proinflammatory cytokines including CXCL1, CXCL10 and MCP-1, but not affecting the level of IL-10 and TGF-β (data not shown). This finding is in line with what has been described in experimental colitis, in which an antibody to the IL-10 receptor did not affect the anti-inflammatory properties of transgenic mice expressing human IL-37 [27]. In our study, we also found that the effects of rhIL-37 were both preventive and therapeutic. In the arthritis model, reduced foot swelling and attenuated pathological joint inflammation were observed in both the rhIL-37 preventive and therapeutic groups.

Third, in the murine acute gouty arthritis model, qPCR-array and western blot analysis indicated that IL-1R8, Smad3 and S​OCS3 were highly expressed, whereas NLRP3 was suppressed in the rhIL-37 intervention groups. These findings suggest that rhIL-37 can suppress MSU-induced inflammation by inhibiting NLRP3 and enhancing expression of IL-1R8 and S​OCS3 for anti-inflammatory signal transduction. Similar results were shown in a murine model of invasive pulmonary aspergillosis [28]. In that study, rhIL-37 markedly reduced NLRP3-dependent neutrophil recruitment and steady-state mRNA levels of IL-1β, and mitigated lung inflammation and damage. Besides NLRP3 inhibition, in our study rhIL-37 also enhanced expression of IL-1R8, Smad3 and S​OCS3.

The anti-inflammatory activity of IL-37 requires IL-1R8 (TIR-8/SIGIRR). Studies by Nold-Petry et al. [4] demonstrate that IL-37 requires the receptors IL-18Rα and IL-1R8 to suppress innate immunity in vitro and in vivo (using the IL-1R8-knockout (KO) mice). In future studies, IL-1R8 silencing and IL-1R8-KO mice studies will be necessary to confirm the contribution of IL-1R8 in rhIL-37 anti-inflammatory properties.

In our study, the expression of Smad3 was also significantly enhanced after rhIL-37 intervention, whereas TGF-β1 was unaffected. This result suggests rhIL-37 may also have an intracellular anti-inflammatory function through interaction with Smad3. Similar results were shown in Marcel’s study [2]. In that study, IL-37 interacted intracellularly with Smad3 and co-localized with phospho-Smad3 to form a functional complex. Furthermore, IL-37-expressing cells and transgenic mice had less cytokine suppression when endogenous Smad3 was depleted. Smad3 inhibition studies are also required in our future research to verify the contribution of Smad3 in rhIL-37 intracellular anti-inflammatory properties.

Chen et al. have shown that mononuclear cells and synovial tissue isolated from the synovium of patients with gout have significantly upregulated expression of the SOCS family proteins cytokine-inducible SH2 protein (CIS) and SOCS3 compared with patients with osteoarthritis. Increased expression of CIS and SOCS3 is observed in MSU-crystal-stimulated human monocyte-derived macrophages, indicating that macrophage activation switches on these regulatory pathways [6]. Our data also support a role for rhIL-37 increased intracellular SOCS3 expression in the resolution of acute gout through the ability to negatively regulate proinflammatory cytokines and to switch on anti-inflammatory cytokine production.

The mRNA levels of Mertk were significantly upregulated in the IL-37 intervention groups. In a previous study by Claudia and colleagues [4], LPS-stimulated THP-1 cells were transfected to express IL-37b, and after inhibition of Mertk the IL-37-mediated anti-inflammation effect was reduced by 81%. In our study, we further demonstrated that Mertk signaling participates in the rhIL-37 inhibitory pathway in gouty models. By inhibition of Mertk, the anti-inflammatory effect of rhIL-37 was partly abrogated, and IL-1R8, Smad3 and SOCS3 were suppressed, whereas NLRP3 expression was reactivated. Previous studies show that Mertk plays a role as an intrinsic feedback inhibitor of the TLR4-driven and inflammatory-mediator-driven immune responses [28–30]. Mertk^-/-^ mice have macrophages deficient in the clearance of apoptotic thymocytes [31] and are found to be characterized with polyclonal lymphocyte proliferation with autoimmune manifestations [32–34]. In a mouse model of LPS-induced acute lung injury, anti-Mertk antibody enhanced LPS-induced inflammatory responses by significantly reducing expression of the TLR suppressors S​OCS1 and S​OCS3. MSU crystals require TLR-2, TLR-4, and MyD88 for macrophage activation [35], and both TLR4- and P2X_7_-dependent (ATP receptor) pathways are required for NLRP3 inflammasome activation [17, 36].

Based on these results and our findings, we hypothesize that Mertk participates in limiting MSU-induced inflammation not only by enhancing macrophage phagocytosis and clearance of MSU crystals and apoptosis of inflammatory cells, but also by inducing signaling in the downregulation of TLR4-activation-driven immune responses by suppressing NLRP3 activation. In this study, the direct link between IL-37 and Mertk, and whether MSU crystals could directly induce Mertk were not demonstrated. However, further study in this area is of great interest.

## Conclusion

In conclusion, our studies strongly indicate that rhIL-37 plays a potent immunosuppressive role in the pathogenesis of both experimental gout models in vitro and in vivo by downregulating proinflammatory cytokines and chemokines, markedly reducing neutrophil and monocyte recruitment, and mitigating pathological joint inflammation. In our studies, rhIL-37 suppressed MSU-induced innate immune responses by enhancing expression of Smad3 and IL-1R8 to trigger multiple intracellular switches to block inflammation, including inhibition of NLRP3 and activation of S​OCS3. Mertk signaling participates in the rhIL-37 inhibitory pathway in gouty models. By inhibition of Mertk, the anti-inflammatory effect of rhIL-37 was partly abrogated, and IL-1R8, Smad3 and S​OCS3 were suppressed, whereas NLRP3 expression was reactivated. Thus, our studies reveal that rhIL-37 limits runaway inflammation initiated by MSU-crystal-induced immune responses, partly in a Mertk-dependent way. Thus, rhIL-37 has both preventive and therapeutic effects in gouty arthritis.

## Abbreviations

AG: Acute gout
AKT: Serine-threonine kinase
AMPK: Adenosine 5‘-monophosphate (AMP)-activated protein kinase
ATF-2: Activating transcription factor-2
CCL-2: chemokine (C-C motif) ligand 2
Ct: cycle threshold
DMEM: Dulbecco’s modified Eagle’s medium
ELISA: Enzyme-linked immunosorbent assay
Erk: Extracellular regulated protein kinase
FACS: Fluorescence-activated cell sorting
FBS: Fetal bovine serum
GAPDH: Glyceraldehyde-3-phosphate dehydrogenase
GSK-3β: Glycogen synthase kinase-3β
H&E: Hematoxylin and eosin
HC: Healthy controls
HRP: Horseradish peroxidase
IL: Interleukin
IP: Interaperitoneally
JNK: Jun N-terminal kinase
LPS: Lipopolysaccharide
Mertk: Mer receptor tyrosine kinase
MLS: macrophage-like synoviocytes
MSU: Monosodium urate
mTOR: Mammalian target of rapamycin
NAG: Non-acute gout
NF-κB: Nuclear factor κB
NLRP3: NACHT-LRR-PYD-containing protein 3
P38MAPK: P38 mitogen-activated protein kinase
PBMC: Peripheral blood mononuclear cells
PBS: Phosphate-buffered saline
PPARγ: Peroxisome proliferator-activated receptor γ
PTEN: Phosphatase and tensin homolog deleted on chromosome ten
qPCR: Quantitative polymerase chain reaction
rhIL: Recombinant human IL
RPMI 1640: Roswell Park Memorial Institute medium
Smad3: Mammalian homologs of the drosophila Mad gene 3
SOCS: Suppressor of cytokine signaling
STAT: Signal transducers and activators of transcription
SYK: Spleen tyrosine kinase
TGF-β1: Transforming growth factor β1
TLR: Toll-like receptor

## References

[1] Dinarello C (2010). IL-1 family nomenclature. Nat Immunol.

[2] Nold MF (2010). IL-37 is a fundamental inhibitor of innate immunity. Nat Immunol.

[3] Bulau AM (2014). Role of caspase-1 in nuclear translocation of IL-37, release of the cytokine, and IL-37 inhibition of innate immune responses. Proc Natl Acad Sci U S A.

[4] Nold-Petry CA (2015). IL-37 requires the receptors IL-18Ralpha and IL-1R8 (SIGIRR) to carry out its multifaceted anti-inflammatory program upon innate signal transduction. Nat Immunol.

[5] Martinon F (2006). Gout-associated uric acid crystals activate the NALP3 inflammasome. Nature.

[6] Steiger S, Harper JL (2014). Mechanisms of spontaneous resolution of acute gouty inflammation. Curr Rheumatol Rep.

[7] Neogi T (2015). 2015 Gout classification criteria: an American College of Rheumatology/European League Against Rheumatism collaborative initiative. Ann Rheum Dis.

[8] Wallace SL (1977). Preliminary criteria for the classification of the acute arthritis of primary gout. Arthritis Rheum.

[9] Denko CW, Whitehouse MW (1976). Experimental inflammation induced by naturally occurring microcrystalline calcium salts. J Rheumatol.

[10] Scanu A (2007). Synoviocyte cultures from synovial fluid. Reumatismo.

[11] Rasool M, Varalakshmi P (2006). Suppressive effect of Withania somnifera root powder on experimental gouty arthritis: an in vivo and in vitro study. Chem Biol Interact.

[12] Post AM (2002). Imaging cell death with radiolabeled annexin V in an experimental model of rheumatoid arthritis. J Nucl Med.

[13] Martin WJ (2011). Monosodium urate monohydrate crystal-recruited noninflammatory monocytes differentiate into M1-like proinflammatory macrophages in a peritoneal murine model of gout. Arthritis Rheum.

[14] Martin WJ, Walton M, Harper J (2009). Resident macrophages initiating and driving inflammation in a monosodium urate monohydrate crystal-induced murine peritoneal model of acute gout. Arthritis Rheum.

[15] Haerdi-Landerer MC, Steiner A, Suter MM (2011). Primary bovine synoviocyte cultures: a useful tool for in vitro drug testing?. Vet J.

[16] Chen H (2016). The effect of resveratrol on the recurrent attacks of gouty arthritis. Clin Rheumatol.

[17] McNamee EN (2011). Interleukin 37 expression protects mice from colitis. Proc Natl Acad Sci U S A.

[18] Xu D (2015). Effects of interleukin-37 on cardiac function after myocardial infarction in mice. Int J Clin Exp Pathol.

[19] Wu B (2014). Interleukin-37 ameliorates myocardial ischaemia/reperfusion injury in mice. Clin Exp Immunol.

[20] Dinarello CA, Bufler P (2013). Interleukin-37. Semin Immunol.

[21] Fujita H (2013). Interleukin-37 is elevated in subjects with atopic dermatitis. J Dermatol Sci.

[22] Zheng SC (2015). Role of the NLRP3 inflammasome in the transient release of IL-1beta induced by monosodium urate crystals in human fibroblast-like synoviocytes. J Inflamm (Lond).

[23] Ruth JH (2010). Expression and function of CXCL16 in a novel model of gout. Arthritis Rheum.

[24] Scanu A (2010). High-density lipoproteins downregulate CCL2 production in human fibroblast-like synoviocytes stimulated by urate crystals. Arthritis Res Ther.

[25] Amaral FA (2012). NLRP3 inflammasome-mediated neutrophil recruitment and hypernociception depend on leukotriene B(4) in a murine model of gout. Arthritis Rheum.

[26] Martinon F (2010). Mechanisms of uric acid crystal-mediated autoinflammation. Immunol Rev.

[27] Imaeda H (2013). Epithelial expression of interleukin-37b in inflammatory bowel disease. Clin Exp Immunol.

[28] Lee YJ (2012). Inhibiting Mer receptor tyrosine kinase suppresses STAT1, SOCS1/3, and NF-kappaB activation and enhances inflammatory responses in lipopolysaccharide-induced acute lung injury. J Leukoc Biol.

[29] Rothlin CV (2007). TAM receptors are pleiotropic inhibitors of the innate immune response. Cell.

[30] O'Neill LA (2007). TAMpering with toll-like receptor signaling. Cell.

[31] Lu Q, Lemke G (2001). Homeostatic regulation of the immune system by receptor tyrosine kinases of the Tyro 3 family. Science.

[32] Cohen PL (2002). Delayed apoptotic cell clearance and lupus-like autoimmunity in mice lacking the c-mer membrane tyrosine kinase. J Exp Med.

[33] Prasad D (2006). TAM receptor function in the retinal pigment epithelium. Mol Cell Neurosci.

[34] Scott RS (2001). Phagocytosis and clearance of apoptotic cells is mediated by MER. Nature.

[35] Liu-Bryan R (2005). Innate immunity conferred by Toll-like receptors 2 and 4 and myeloid differentiation factor 88 expression is pivotal to monosodium urate monohydrate crystal-induced inflammation. Arthritis Rheum.

[36] Higashimori A (2016). Mechanisms of NLRP3 inflammasome activation and its role in NSAID-induced enteropathy. Mucosal Immunol.

